# Development and validation of an electronic health record 3‐year risk model for diagnosis of dementia with and without Mini‐Cog scores

**DOI:** 10.1002/alz.092569

**Published:** 2025-01-09

**Authors:** Leah R Hanson, A Lauren Crain, Sally K Gustafson, Ann M Werner, Aleta L Svitak, Lauren R O'Keefe, Patrick J O'Connor, Rebecca C Rossom

**Affiliations:** ^1^ HealthPartners Institute, Minneapolis, MN USA; ^2^ HealthPartners Center for Memory & Aging, Saint Paul, MN USA

## Abstract

**Background:**

The number people living with Alzheimer’s disease and related dementias is expected to triple by 2050, contributing to decreased quality of life, increased medical care utilization, and additional burden on an already stressed primary care system. Many clinicians lack confidence to assess, diagnose and manage cognitive impairment (CI), and more than 50% of patients with CI are undiagnosed. A tool to better identify patients at risk of CI could lead to earlier detection and diagnosis in primary care.

**Method:**

A prediction model was developed and validated to estimate the risk of dementia diagnosis within three years using two machine learning approaches, R SuperLearner and LASSO. Patients were 65 years or older without a CI diagnosis and seen at a 2017 primary care index visit. A subgroup of patients had Mini‐Cog (MC) screening data available. Variable selection was carried out in the full population, and again in the MC subgroup. Both machine learning approaches selected variables from >1,000 features calculated from broad categories of electronic health record (EHR) and MC data documented between 2012 and the index date. Features considered came from categories including Annual Wellness Questionnaire, cognitive screens, diagnoses, encounter history, vitals, labs, medications, procedures, patient reported outcomes, social history, and vaccinations. Dementia diagnosis between index and 12/31/2020 was the outcome. Diagnostic measures were calculated from logistic regression models fit using the selected variables.

**Result:**

The full population prediction model included data from N = 100,998 patients (training n = 70,699; validation n = 30,299), 6.0% of whom developed dementia. In the MC subgroup (training n = 47,478; validation n = 20,807) 3.6% developed dementia. Maximum predictive accuracy (AUC) was obtained using the LASSO approach. The full population model retained 33 predictors with AUC = 0.80 (95% CI 0.79 – 0.81) in the training and validation datasets. The MC prediction model retained 32 predictors and AUC = 0.83 (95% CI 0.82‐0.84) in both datasets.

**Conclusion:**

Using MC and readily available EHR data, we were able to predict future dementia diagnoses with high accuracy. Next, we are implementing this model into an algorithm‐driven clinical decision support system and testing the system’s ability to improve diagnosis rates via a cluster‐randomized trial.

